# Supplementary material for: Metformin enhances anti-mycobacterial responses by educating CD8+ T-cell immunometabolic circuits
Source: Nat Commun. 2020 Oct 16;11:5225. doi: 10.1038/s41467-020-19095-z (PMC7567856; doi:10.1038/s41467-020-19095-z)
Supplement: Supplementary file 1 — Supplementary Information [file 41467_2020_19095_MOESM1_ESM.pdf]

Title: Metformin enhances anti-mycobacterial responses by educating immunometabolic circuits of $CD8^+$ T cells

Böhme et al.

Supplemental Methods

Analysis of CyTOF data. Samples for each dual barcode combination were debarcoded manually using FlowJo software (Treestar. Inc). Analysis of live gated mouse $CD8^+$ T cells (fig. S2A) or live gated human $CD8^+$ T cells (fig. S7A) was carried out using the t-SNE (t-Distributed Stochastic Neighbor Embedding) dimension reduction. Phenograph was used to cluster subpopulations according to phenotype relatedness in high-dimensional single-cell data using Euclidean distance (*57*). For automated clustering, we performed k-means clustering of the t-SNE output, using 10 centres and 1,000 random repeats. The $\chi 2$-test was used to assess the correlation between the two grouping methods. In R, all data were transformed using the "logicleTransform" function by using the "flowCore" package (parameters: w = 0.25, t = 16,409, m = 4.5, a = 0). We calculated the percentages and median intensity values for each marker assessed and used heat maps to show intensities of markers expression (*23*). t-SNE derived heat maps were generated by using customized R-scripts. Statistical analysis was done using two-tailed paired *t*-test. $p < 0.05$ was considered significant.

Quantitative RT-PCR primers used in the study. The following primers were used: *Gapdh* forward 5'-TCGTCCCGTAGACAAAATGG-3', *Gapdh* reverse 5'-TTGAGGTCAATGAAGGGGTC-3', *Cxcr3* forward 5'-CCCAACCACAAGTGCCAAAG-3', *Cxcr3* reverse 5'-TCACTAACCTCAAGGTACATGGC-3', *Cxcl9* forward 5'-TCGGACTTCACTCCAACACAG-3', *Cxcl9* reverse 5'-AGGGTTCCTCGAACTCCACAC-3', *Cxcl10* forward 5'-GCCGTCATTTTCTGCCTCA-3', *Cxcl10* reverse 5'-CGTCCTTGCGAGAGGGATC-3', *Cxcl11* forward 5'-CAGCTGCTCAAGGCTTCCTTA-3', *Cxcl11* reverse 5'-CTTTGTCGCAGCCGTTACTC-3', *Sell* forward 5'-GGGGAGCCCAACAACAAGAA-3', *Sell* reverse 5'-CACTGGACCACTGTGTAGCA-3', *Cpt1a* forward 5'-CCGGAAAGGTATGGCCACTT-3', *Cpt1a* reverse 5'-GAAGAAAATGCCTGTCGCCC-3', *Tfam* forward 5'-CTGCTCTTTATACTTGCTCACAG-3', *Tfam* reverse 5'-CACCCAGATGCAAAACTTTCAG-3'.

Supplemental Figures

a. Gating Strategy for Exp 1 (CD45.2 ➜ irradiated CD45.2)

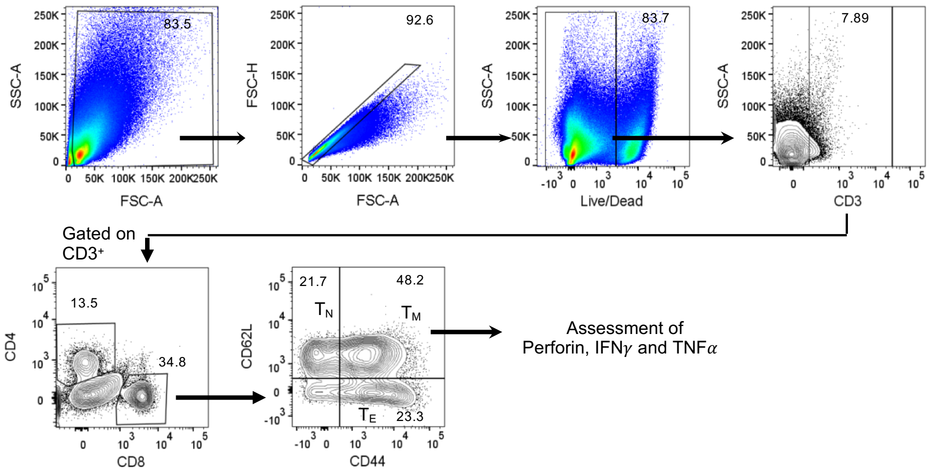

b. Number of total CD3+, CD4+ and CD8+ T cells (Exp 1)

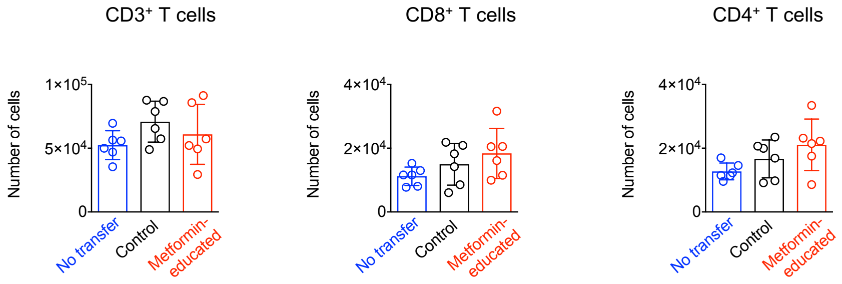

c. Gating Strategy for Exp 2 (CD45.1 ➜ irradiated CD45.2)

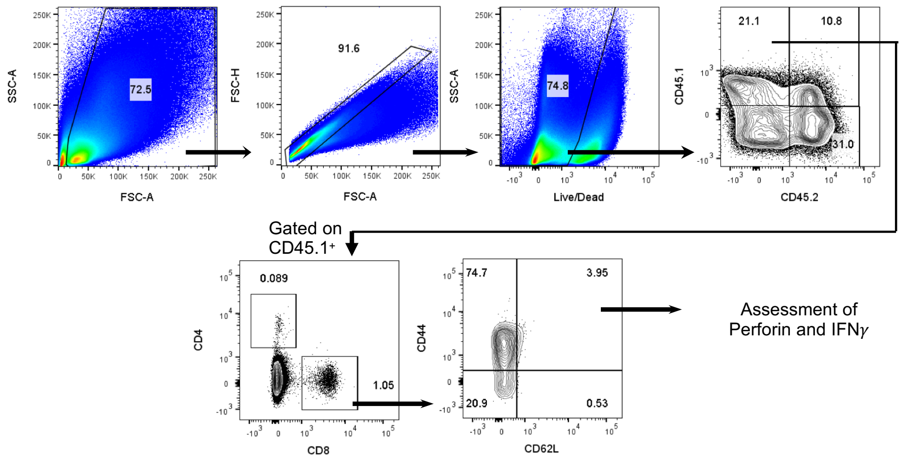

Supplementary Fig 1. Phenotyping of Metformin-educated CD8$^+$ T cell from naïve wildtype mice in spleen during *Mtb* infection.

a Flow cytometry gating strategy to analyse spleen cells of recipient mice in Exp 1, used in Fig. 1d-f. **b** Flow cytometry analysis of splenic T cell populations described in Fig. 1d-f during the course of *Mtb* infection. Absolute numbers of CD3$^+$, CD8$^+$ and CD4$^+$ T cells show no difference between the three groups. Results shown as mean ± SD (n = 6). **c** Flow cytometry gating strategy to analyse spleen cells of recipient mice in Exp 2, used in Fig. 1g.

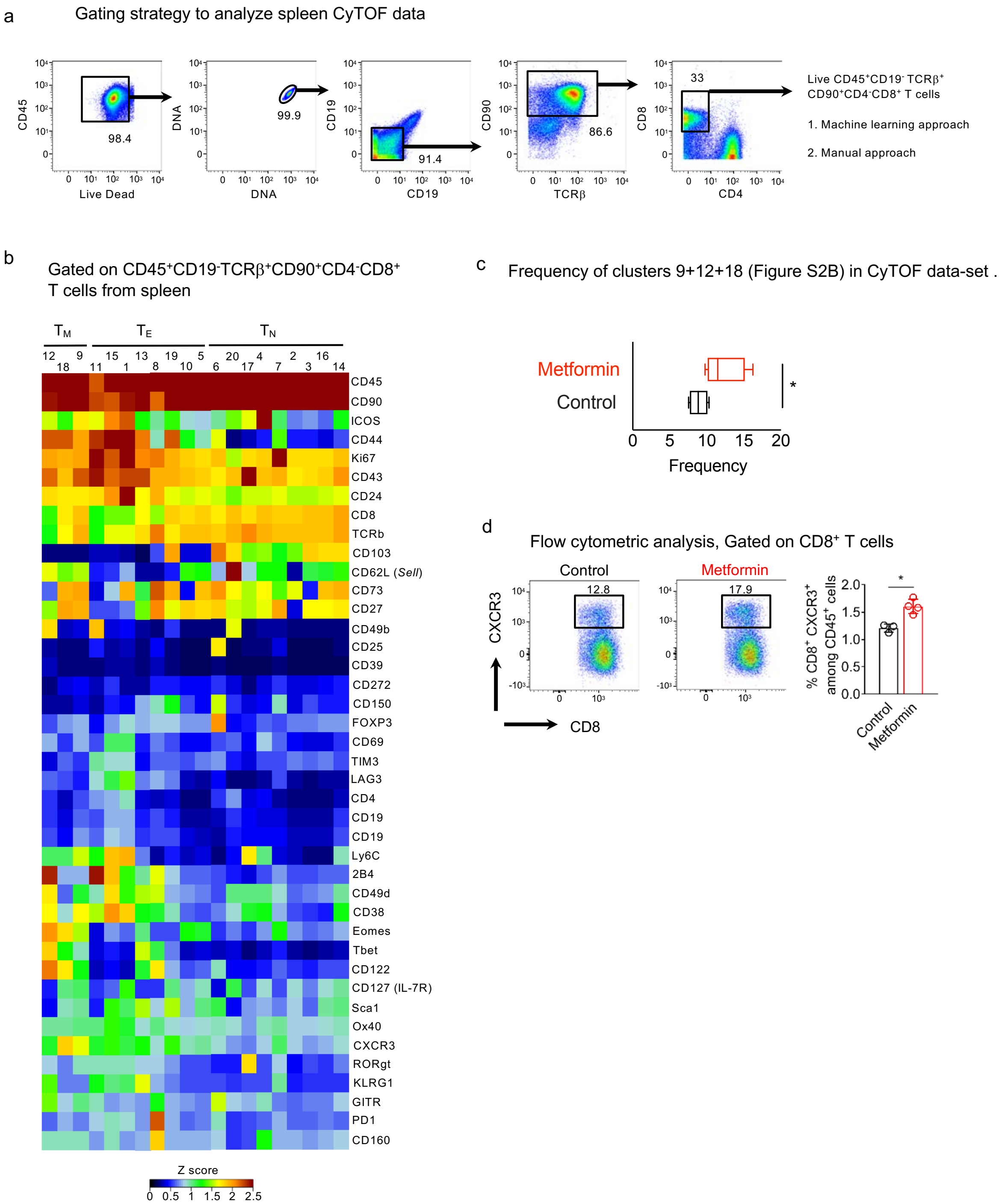

Supplementary Fig 2. Metformin-treatment expands cluster of CD8^{+} T$_{M}$ cells and CXCR3 expressing CD8^{+} T cells in the spleen of wild type mice.

a Gating strategy used for phenograph based automated machine learning and manual analysis of splenic CD8^{+} T cells. **b** Heat map of the respective median expression for all stained markers among 20 clusters

identified by phonograph in splenic $CD8^+$ T cells. Clusters are divided into those belonging to T_M, T_E and T_N cells. Data from 4 mice per group. Results are shown as z score. **c** Machine-learning automatic gating identified three cluster (9, 12, 18) corresponding to $CD8^+$ T_M cells. Data shown is a frequency of three clusters among two groups. *p=0.05, two-tailed Mann-Whitney *U* test. Box-and-whisker plot show the median, 5 and 95 percentiles. **d** Flow cytometric analysis of splenic $CD8^+$ T cells expressing CXCR3 in control and metformin-treated WT mice. Data shown one out of two experiments, mean ± SD of 3 -4 mice per group, *p=0.05, two-tailed Mann-Whitney *U* test.

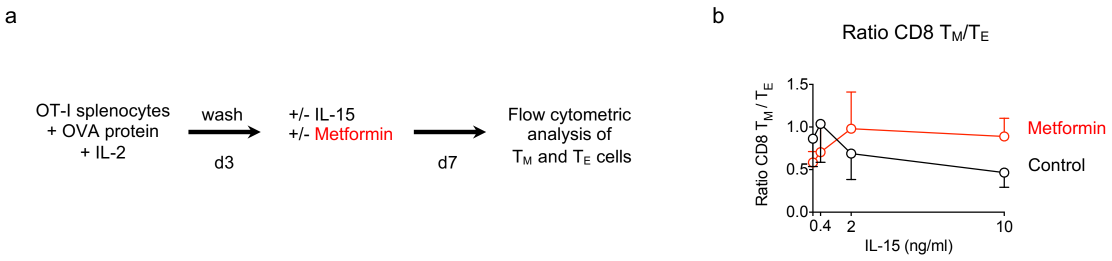

Supplementary Fig 3. *In vitro* treatment with metformin increases ratio of Ag-specific CD8$^+$ T_{CM} over T_E.

a Experimental set-up of *in vitro* experiment. Splenocytes of OT-I mice were isolated and activated with Ova protein and IL-2 for 3 days. After washing and media exchange, Ag-specific splenocytes were cultured for additional 4 days with different concentrations of IL-15 in the presence or absence of metformin. After this cells were analysed by flow cytometry for the expression of CD44 and CD62L markers. **b** CD8$^+$ T_{CM}/T_E ratio was analysed. Combined data of two independent experiments is shown, means ± SEM of 4 mice/ group.

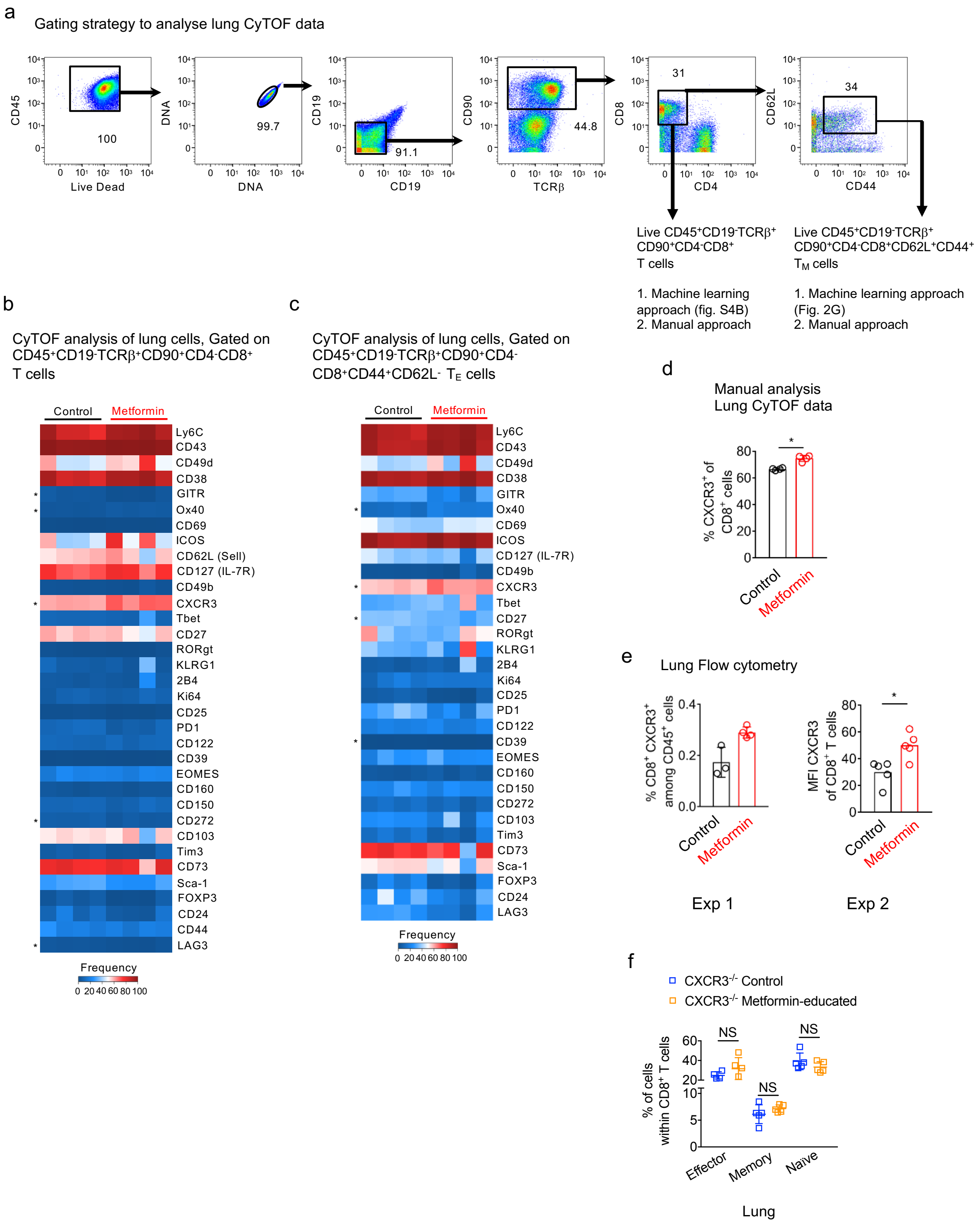

Supplementary Fig 4. Metformin-treatment increases CXCR3 expression in the lung CD8⁺ T cells and their subsets from wild type mice

a Gating strategy used for Phenograph based automated machine learning and manual analysis of lung $CD8^+$ T and $CD8^+$ T_M cells. **b,c** Heat map of the respective median expression for all stained markers in lung $CD8^+$ T (B) and $CD8^+$ T_E (C) cells. Data shown from 4 mice per group, *$p = 0.04$, two-tailed paired *t* test. **d** Manual analysis of CyTOF data to characterize CXCR3 expression on lung $CD8^+$ T cells. Data shown from 4 mice. *p=0.028, two-tailed Mann-Whitney *U* test. **e** Flow cytometric analysis of the frequency of lung $CD8^+CXCR3^+$ T cells in control and metformin-treated wild type mice. MFI of CXCR3 among $CD8^+$ T cells is also depicted. Data shown from 5 mice. *p=0.031, two-tailed Mann-Whitney *U* test. **f** Flow cytometric analysis of lung $CD8^+$ T_E, T_M, and T_N cells of control and metformin-treated *CXCR3*$^{-/-}$ mice. n=4 mice/group. Data shown from one out of two experiments.

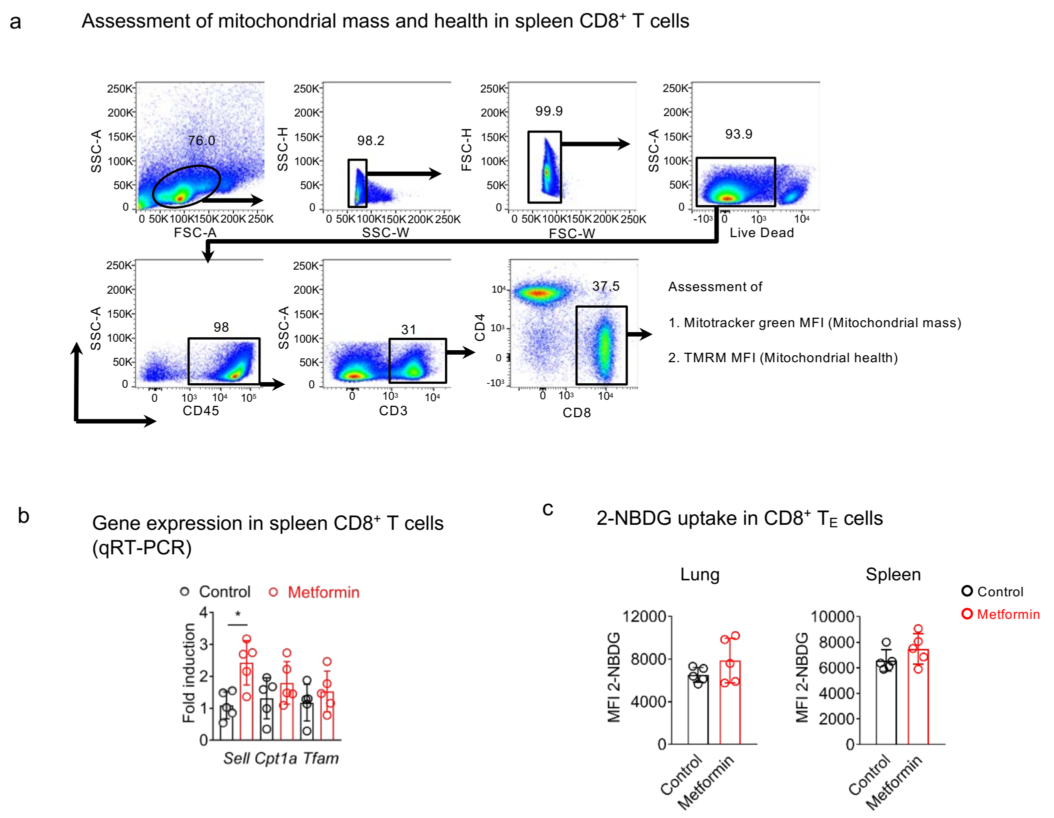

Supplementary Fig 5. Assessment of mitochondrial mass and health, and glucose uptake in CD8$^+$ T cells.

a Gating strategy to assess changes in mitochondrial mass and membrane potential in splenic CD8$^+$ T cells of wild type mice treated or not with metformin. **b** Expression of *Sell* (CD62L), *Cpt1a* and *Tfam* mRNA in the splenic CD8$^+$ T cells was measured using qRT-PCR, in metformin-treated and untreated (control) mice. Gene expression was normalized to *Gapdh* expression and fold induction was calculated with respect to untreated CD8$^+$ T cells (control). Results shown as mean ± SD (n = 5 mice) from one representative experiment, $*p$=0.03, two-tailed Mann-Whitney *U* test. **c** Lung and spleen CD8$^+$ T cells were starved of glucose, following which 2-NBDG (glucose analogue) uptake was measured in memory and effector CD8$^+$ T cells. The data (MFI) shown is of CD8$^+$ T_E cells, mean ± SD (n = 5 mice). Data from one out of two independent experiment shown.

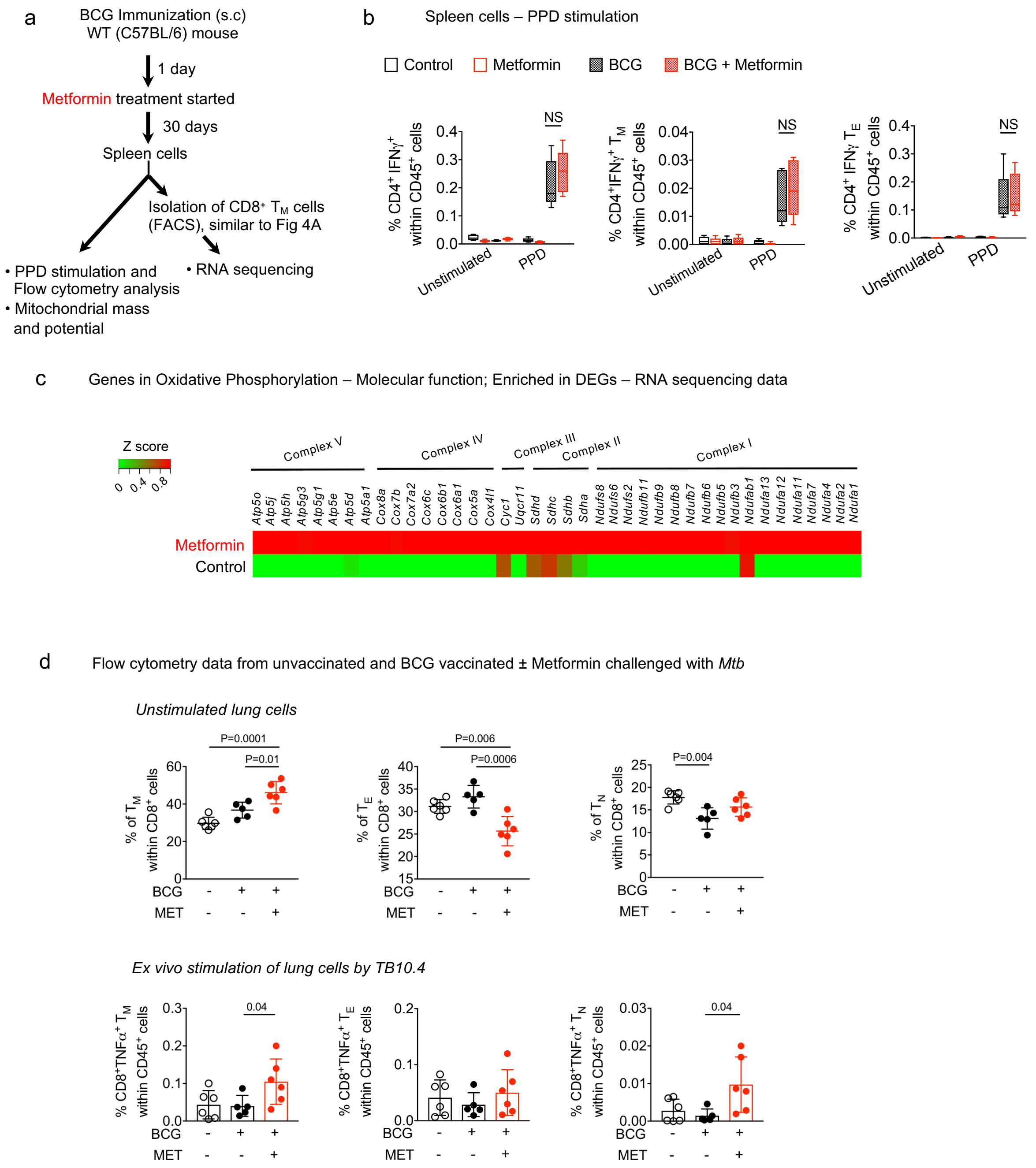

Supplementary Fig 6. Metformin reprograms host oxidative phosphorylation and immune response in BCG vaccinated mice.

a Schematic of metformin-treatment of BCG-vaccinated mice. WT mice were vaccinated with BCG *s.c.* and on following day fed with metformin in drinking water for 30 days. At 30 days spleen cells were characterized for (i) cellular phenotyping via flow cytometry, and (ii) response to PPD. $CD8^+$ T cells were

also isolated and gene expression analysis (RNA sequencing) was carried out. **b** PPD-stimulated splenocytes were assessed *ex vivo* for IFNγ-producing $CD4^+$ T, T_M and T_E cells. Box-and-whisker plot show the median, 5 and 95 percentiles. n = 5 mice/group. Data from one out of two independent experiments are shown. Statistical analysis was performed by ANOVA (NS not significant). **c** Molecular function enrichment (by IPA) of DEGs (obtained by analysing RNA seq data) in $CD8^+$ T_M cells from metformin-treated BCG-vaccinated mice compared with untreated BCG-vaccinated mice. Heat map of 44 electron transport genes (different complexes) for the enriched oxidative phosphorylation ontology (OXPHOS, Fig. 5d) is being shown. All of the OXPHOS genes were upregulated in the $CD8^+$ T_M cells from metformin-treated BCG-vaccinated mice. **d** Flow cytometry analysis of splenic T cell populations of animals described in Fig. 5e, during the course of *Mtb* infection. *Above panel* - Frequency of $CD8^+$ T_M, T_E and T_N cells are shown for three groups. *Bottom panel* - TB10.4 specific response of spleen cells. Data on TNFα producing $CD8^+$ T_M, T_E and T_N cells from one out of two independent experiments is shown. Mean ± SD (n = 5 mice/group). One-way ANOVA with Turkey's multiple comparison test.

a Gating strategy to analyze healthy donor CyTOF data

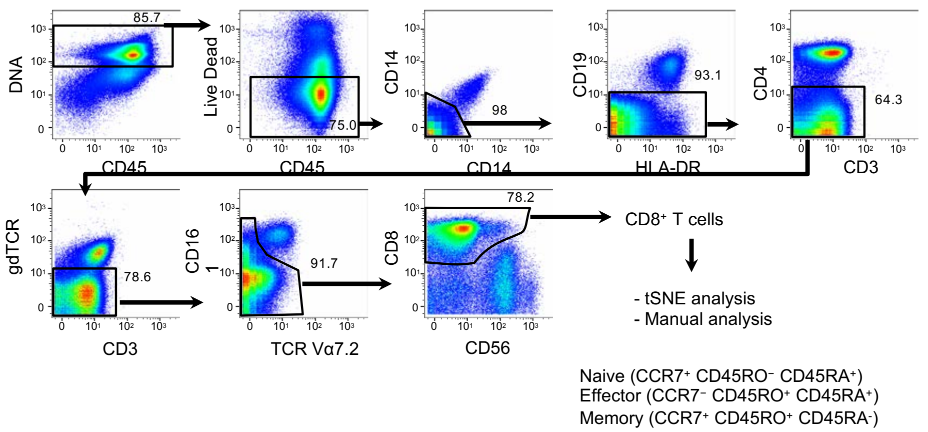

b Gating strategy to analyze DM patients' flow cytometry data

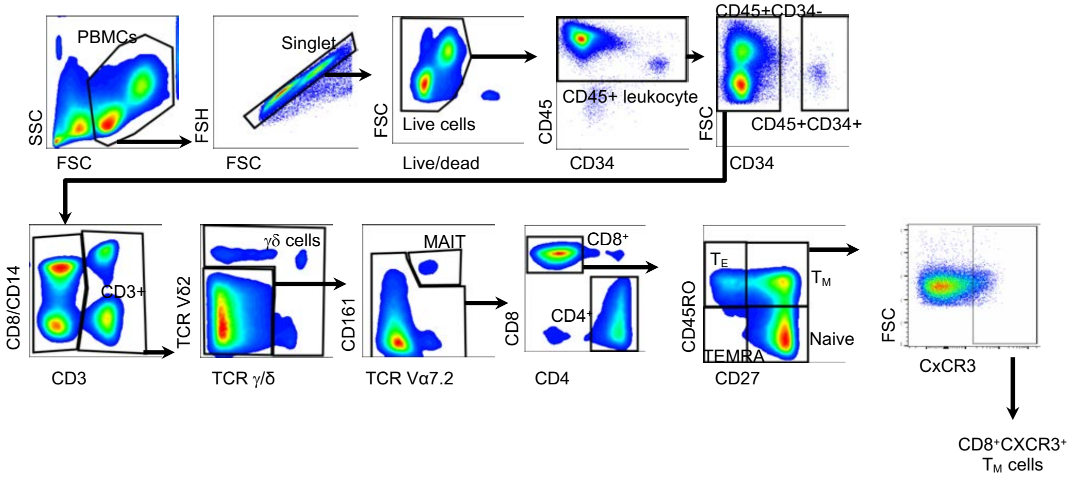

Supplemental Figure 7. Effect of metformin treatment on the phenotype of peripheral CD8$^+$ T cells from healthy and T2D individuals.

a Strategy to gate peripheral CD8$^+$ T cells in CyTOF data from healthy individuals to perform phonograph based t-SNE and manual analysis. Marker characteristics of T_M, T_E and T_N cells is shown. **b** Gating strategy to analyse PBMCs of T2D patients for the expression of CXCR3 on peripheral CD8$^+$ T_M cells.

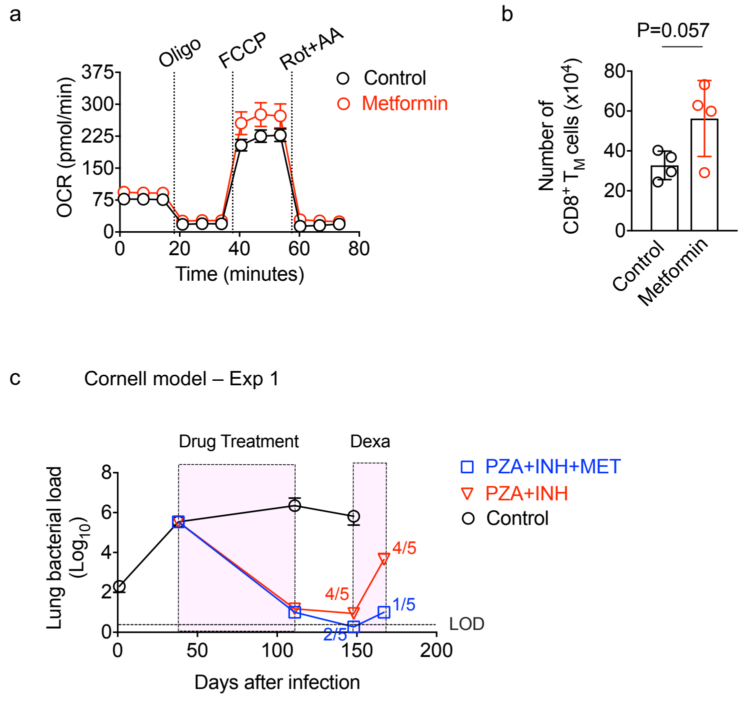

Supplemental Figure 8. Effect of metformin on *Mtb* clearance and reactivation.

a Oxygen consumption rate (OCR) in spleen CD8$^+$ T cells from control and metformin-treated (6 months) WT mice in response to different drugs. Means ± SD of 5 mice per group**.** Data from one experiment. **b** Number of splenic CD8$^+$ T_M cells in mice treated with metformin for 6 months, n=4. P=0.057, Mann-Whitney *U* test. **c** Lung bacillary load in Cornell Model (Exp 1) at respective time points post-infection (p.i) is shown in mice treated with various drug combinations. Time period of drug treatment, rest and dexamethasone (Dexa) treatment has been indicated. Number of mice with detectable *Mtb* in the lung at day 150 p.i and day 165 p.i has been depicted. PZA+INH – mice treated with pyrazinamide and isoniazid; PZA+INH+MET – mice treated with pyrazinamide, isoniazid and metformin; control – No drug treatment. n=3-5 per group/timepoint.

Supplementary Tables

Supplementary Table 1. Mouse antibodies used for mass cytometry analysis listing metal conjugate, antibody clone, supplier of each marker.

Isotope Tag	Antibody	Clone	Company	Catalogue nb
Y-89	CD45	30-F11	Fluidigm	3089005B
Qdot Cd112/114	CD19	6D5	Life technologies	Q10379
Pd-102	Barcode			
Rh-103	Barcode			
Pd-104	Barcode			
Pd-105	Barcode			
Pd-106	Barcode			
Pd-108	Barcode			
Pd-110	Barcode			
In-113	Barcode			
In-115	CD90	T24/31	BioXcell	BE0076
Ce-140	Ly6C	HK1.4	Biolegend	128002
Pr-141	CD43	S11	Biolegend	143202
Nd-142	TCR-beta	H57-597	Biolegend	109202
Nd-143	CD49d	R1-2	Biolegend	103701
Nd-144	CD38	90	Biolegend	102702
Nd-145	GITR	DTA-1	Biolegend	126323
Nd-146	CD8a	53-6.7	Biolegend	100702
Sm-147	OX40	OX-86	Biolegend	119430
Nd-148	CD69	H1.2F3	Invitrogen	14-0691-82
Sm-149	CD4	H129.15	Biolegend	130302
Nd-150	ICOS	C398.4A	Biolegend	313502
Eu-151	CD62L	MEL-14	Biolegend	104402
Sm-152	CD127	A7R34	Biolegend	135002
Eu-153	CD49b	DX5	Biolegend	108902

Sm-154	CXCR3	CXCR3-173	Biolegend	126502
Gd-155	Tbet	4B10	Biolegend	644801
Gd-156	CD27	LG.7F9	eBioscience	14-0271-82
Gd-157	Ror-gamma T	B2D	eBioscience	14-6981-82
Gd-158	KLRG-1	2F1	BD	562190
Tb-159	2B4	M2B4(B6)458.1	Biolegend	133501
Gd-160	Ki67	B56	BD	556003
Dy-161	CD25	PC61	Biolegend	102002
Dy-162	PD-1	29F.1A12	Biolegend	135202
Dy-163	CD122 APC	TM-b1	eBioscience	14-1222-82
Dy-164	CD39	5F2	Biolegend	135702
Ho-165	Eomes	Dan11mag	eBioscience	14-4875-82
Er-166	CD160	7H1	Biolegend	143002
Er-167	CD150 PE	TC15-12F12.2	Biolegend	115903
Er-168	CD272	6A6	Biolegend	139118
Tm-169	CD103	2E7	Biolegend	121402
Er-170	TIM-3	B8.2C12	Biolegend	134002
Yb-171	CD73	TY/23	BD	550738
Yb-172	Sca-1	D7	Biolegend	108102
Yb-173	FOXP3	MF-14	Biolegend	126402
Yb-174	CD24	M1/69	Biolegend	101802
Lu-175	CD44	IM7	BioXcell	BE0039
Yb-176	Lag-3	C9B7W	Biolegend	125202
Ir-191/193	DNA		Fluidigm	201192A
Pt-195	CisPlatin live/dead		Sigma	201064

Supplementary Table 2: Enrichment of Canonical Pathways (IPA analysis) in 202 DEGs between splenic CD8+ T cells from CXCR3-KO vs WT mice.

S.No	Category	log (p value)	Molecules
1	Communication between Innate and Adaptive Immune Cells	7.88	CCL3L3,CCL4,CD83,HLA-A,IFNG,Igha,Ighg2b,IGHM,IL1B
2	IL-7 Signaling Pathway	7.37	FOXO1,GSK3B,IFNG,Igha,Ighg2b,IGHM,JUN,MYC
3	Systemic Lupus Erythematosus In B Cell Signaling Pathway	6.58	FASLG,FOS,FOXO1,GSK3B,IFNG,IGHM,IL1B,JUN,MYC,RRAS2,TGFB1,TNFSF14
4	Thyroid Cancer Signaling	6.07	CXCR4,FOS,FOXO1,GSK3B,JUN,MYC,RRAS2
5	Hepatic Fibrosis Signaling Pathway	6.04	FOS,FOXO1,FTL,GSK3B,IL1B,IL1R2,JUN,MYC,NFKBID,RHOB,RRAS2,TF,TGFB1
6	HMGB1 Signaling	5.85	FASLG,FOS,IFNG,IL1B,JUN,RHOB,RRAS2,TGFB1,TNFSF14
7	Atherosclerosis Signaling	5.75	CCR2,CXCR4,IFNG,IL1B,LYZ,S100A8,TGFB1,TNFSF14
8	Production of Nitric Oxide and Reactive Oxygen Species in Macrophages	5.39	FOS,IFNG,IRF8,JUN,LYZ,NFKBID,PPP2R2A,RHOB,S100A8
9	IL-10 Signaling	5.23	CCR5,FOS,IL1B,IL1R2,JUN,NFKBID
10	Chemokine Signaling	4.85	CCL4,CCR5,CXCR4,FOS,JUN,RRAS2
11	Role of Osteoblasts, Osteoclasts and Chondrocytes in Rheumatoid Arthritis	4.84	FOS,FOXO1,GSK3B,IFNG,IL1B,IL1R2,JUN,NFKBID,TGFB1
12	T Cell Exhaustion Signaling Pathway	4.7	FOS,FOXO1,HLA-A,IFNG,JUN,PPP2R2A,RRAS2,TGFB1
13	Pathogenesis of Multiple Sclerosis	4.66	CCL4,CCR5,CXCR3
14	IL-12 Signaling and Production in Macrophages	4.59	FOS,IFNG,IRF8,JUN,LYZ,S100A8,TGFB1
15	Neuroinflammation Signaling Pathway	4.55	CX3CR1,FASLG,FOS,GSK3B,HLA-A,IFNG,IL1B,JUN,TGFB1,TYROBP
16	Hepatic Cholestasis	4.53	FASLG,IFNG,IL1B,IL1R2,JUN,NFKBID,TGFB1,TNFSF14
17	Role of Macrophages, Fibroblasts and Endothelial Cells in Rheumatoid Arthritis	4.4	FOS,GSK3B,Ighg2b,IL1B,IL1R2,JUN,MYC,NFKBID,RRAS2,TGFB1
18	Aryl Hydrocarbon Receptor Signaling	4.36	ALDH2,FASLG,FOS,IL1B,JUN,MYC,TGFB1
19	Colorectal Cancer Metastasis Signaling	4.36	FOS,GSK3B,IFNG,JUN,MMP15,MYC,RHOB,RRAS2,TGFB1

20	PPAR Signaling	4.21	FOS,IL1B,IL1R2,JUN,NFKBID,RRAS2
21	ErbB2-ErbB3 Signaling	4.18	FOXO1,GSK3B,JUN,MYC,RRAS2

Supplementary Table 3: Enrichment of molecular functions (IPA analysis) in 267 DEGs between splenic memory-like CD8+ T cells from metformin-treated vs untreated mice.

	Category	p value	Molecules
1	Cell Death and Survival	4.05E-08-1.07E-02	KDM6B,CD3E,KLRB1,LIPE,FOXP3,MIF4GD,HBA1/HBA2,C8orf44-SGK3/SGK3, BRCC3,ELOC, TOP1,Gm21596/Hmgb1,ANXA11,Rpl23a,SUB1,Map3k7,TDP2,FOSL1,KAT2A,HK1,MYO6,DPF2,SH3BP2,SLC17A5,HELLS,Abcb1b,NLRX1,SSRP1, AGAP2,MACO1,FLOT2,ARHGAP18,DCLRE1C,CAPN1,CREM,HNRNPUL2,PSMA4,HSPB11,TBC1D15,RPS6KA1,PDCD5,SNCA,NRP1,AKAP12,Calm1 (includes others),NACC2, NUDT2,NUAK2,KSR1, PDCD4,CCT4,TGM2,HMOX1,MTOR,NOTCH2,Ppp1cc,NFYA,CASP1,PSMA3,STIM1,Pvr,VASP,ZNF274,PRMT2,GZMK,ASB2,BEX3,CCHCR1,GPR132,ANXA4,PLK1,PDLIM4,MSI2,ITGAL,HBB,DUSP14,BAK1,XPA,CDK1,ROCK1,ATP6AP2,IMMT,WAS,ICOS,PRMT5,RPS27A,IL2RA,CYCS,C
2	Cellular Development	5.75E-05-1.07E-02	KDM6B,CD3E,FMR1,FOXP3,HBA1/HBA2,C8orf44-SGK3/SGK3,ZBTB7B,TOP1,Gm21596/Hmgb1, Map3k7,FOSL1,KAT2A,HK1,CDK13,AGAP2,MR1,IFITM3,CCNG2,DCLRE1C,FSCN1,CREM,PSMA4,RPS6KA1,PIP4K2A,SNCA,NRP1,AKAP12,RALA,NACC2,HNRNPA2B1,PDCD4,TGM2,HMOX1,MTOR,NOTCH2,Ppp1cc,RASAL1,NFYA,CHN2,CASP1,STIM1,VASP,ASB2,PLK1,PDLIM4,CISD2,ITGAL,CDK1,BAK1,IMMT,WAS,IFT20,TTC7A,ICOS,PRMT5,CYCS,IL2RA,UBE2D3
3	Cellular Function and Maintenance	5.75E-05-1.07E-02	C9orf72,CD3E,SIDT2,FMR1,KLRB1,LIPE,FOXP3, HEBP1,ZBTB7B,Gm21596/Hmgb1,ESPN,Map3k7, MYO6,ACTR2,SH3BP2,SLC17A5,NLRX1,DCLRE1C,ARHGAP18,FSCN1,CAPN1,SNCA,PIP4K2A, NRP1,AKAP12,RALA,NUAK2,SPTSSA,TGM2,HMOX1,MTOR,NOTCH2,CASP1,STIM1,VASP,GZMK,GPR132,PDLIM4,CISD2,ITGAL,BAK1,CDK1.ROCK1.IMMT.WAS.ICOS.TTC7A.IL2RA.CYCS

4	Cellular Growth and Proliferation	5.75E-05-1.07E-02	IFG,CD3E,KLRB1,FOXP3,C8orf44-SGK3/SGK3,HBA1/HBA2,ZBTB7B,TOP1,Gm21596/Hmgb1,ANXA11,URGCP,Map3k7,FOSL1,KAT2A,AHCY,HK1,ATP5MC2,CDK13,BIN3,SH3BP2,Abcb1b,NLRX1,AGAP2,MR1,IFITM3,CCNG2,FLOT2,DCLRE1C,CAPN1,FSCN1,CREM,PSMA4,RPS6KA1,PIP4K2A,PDCD5,SNCA,NRP1,AKAP12,NACC2,RALA,HNRNPA2B1,GBF1,KSR1,PDCD4,TGM2,HMOX1,MTOR,NOTCH2,RASAL1,NFYA,CHN2,CASP1,RNH1,Pvr,STIM1,VASP,ZNF274,GZMK,CKS2,ASB2,CCHCR1,BEX3,GPR132,PLK1,PDLIM4,MSI2,CISD2,ITGAL,XPA,DUSP14,BAK1,CDK1,IL18RAP,ROCK1,IMMT,USP4,WAS,KIF20B,TTC7A,ICOS,PRMT5,CYCS,IL2RA,UBE2D3
5	Cellular Assembly and Organization	1.37E-04-1.07E-02	AKAP12,RALA,FMR1,LIPE,NUAK2,DYNLT1,TGM2,MTOR,TOP1,ESPN,STIM1,MTMR2,VASP,MYO6,HK1,ACTR2,GZMK,SLC17A5,PLK1,HELLS,SGCB,PDLIM4,SSRP1,BAK1,CDK1,ROCK1,CCNG2,ARHGAP18,WAS,KIF20B,CAPN1,FSCN1,ICOS,CYCS,SNCA,NRP1
6	Cell-To-Cell Signaling and Interaction	1.49E-04-1.07E-02	RALA,CD3E,FMR1,KLRB1,HNRNPA2B1,FOXP3,KSR1,TGM2,ZBTB7B,HMOX1,NOTCH2,MTOR,Gm21596/Hmgb1,CASP1,Map3k7,STIM1,MTMR2,ASB2,GPR132,SH3BP2,ITGAL,BAK1,IL18RAP,MR1,ROCK1,WAS,ICOS,CYCS,IL2RA,SNCA,NRP1
7	Cell Morphology	1.85E-04-1.07E-02	AKAP12,RALA,CD3E,C9orf72,FMR1,HNRNPA2B1,FOXP3,LIPE,GBF1,SPTSSA,TGM2,HMOX1,MTOR,Gm21596/Hmgb1,ESPN,CASP1,Map3k7,STIM1,FOSL1,VASP,MYO6,HK1,GZMK,ASB2,CCHCR1,GPR132,SGCB,PLK1,NLRX1,ITGAL,CISD2.CDK1.BAK1.XPA.ROCK1.ATP6AP2.IMMT.W
8	Cell Cycle	3.4E-04-1.07E-02	AKAP12,RALA,CD3E,IST1,GBF1,C8orf44-SGK3/SGK3,BRCC3,ELOC,CCT4,HMOX1,NOTCH2,TOP1,MTOR,NFYA,CHN2,UBL5,FOSL1,Pvr,KAT2A,VASP,ZNF274,CDK13,CKS2,GPR132,SH3BP2,HELLS,PLK1,SSRP1,ITGAL,XPA,CDK1,BAK1,CCNG2,WAS,KIF20B,CREM,ICOS,PRMT5,COP1,SNCA,UBE2D3,NRP1

9	Cellular Movement	3.4E-04-1.07E-02	KDM6B,CD3E,LIPE,FOXP3,HEBP1,C8orf44-SGK3/SGK3,Gm21596/Hmgb1,TDP2, FOSL1, AHCY,MYO6,ACTR2,Abcb1b,AGAP2,SSRP1,IFITM3,CAPN1,FSCN1,RPS6KA1,SNCA,NRP1,AKAP12,RALA,IST1,HNRNPA2B1,GBF1,NUAK2,PDCD4,TGM2,HMOX1,NOTCH2,MTOR,CHN2,CASP1,RNH1,STIM1,Pvr,VASP,PRMT2,ASB2,GPR132,SLIRP,PLK1,PDLIM4,ITGAL,CDK1,ROCK1,USP4,WAS,KIF20B,ICOS,PRMT5,IL2RA,UBE2D3
10	Cellular Compromise	5.3E-04-1.07E-02	RALA,KLRB1,GPR132,FOXP3,SH3BP2,PILRA,GBF1,ITGAL,XPA,TGM2,HMOX1,NOTCH2,WAS,ICOS,CREM,CASP1,Map3k7,STIM1,IL2RA,SNCA,KAT2A
11	DNA Replication, Recombination, and Repair	6.1E-04-1.07E-02	PAXX,CKS2,FOXP3,PLK1,BRCC3,SSRP1,CDK1,XPA,HMOX1,MTOR,TOP1,Gm21596/Hmgb1,DCLRE1C,SUB1,TDP2,IL2RA

Supplementary Table 4. Enrichment of molecular functions (IPA analysis) in 607 DEGs between splenic memory-like CD8+ T cells from metformin-treated BCG-vaccinated vs untreated BCG-vaccinated mice.

	Category	-log(p value)	Ratio	Molecules
1	Oxidative Phosphorylation	32.2	0.321	NDUFA4,COX7B,ATP5MC1,ATP5PD,COX6A1,NDUFA7,ATP5F1D,Cox6c,NDUFB5,COX8A,NDUFB8,NDUFA1,NDUFB3,NDUFA2,Atp5e,NDUFB9,NDUFS6,NDUFS2,NDUFB6,ATP5MC3,COX4I1,ATP5PF,COX6B1,ATP5F1A,ATP5PO,UQCR11,NDUFA13,NDUFB11,NDUFS8,NDUFA11,UQCR10,NDUFB7,COX7A2,COX5A,NDUFA12
2	Mitochondrial Dysfunction	26	0.211	NDUFA4,ATP5MC1,COX7B,ATP5PD,COX6A1,NDUFA7,ATP5F1D,Cox6c,NDUFB5,COX8A,NDUFB8,NDUFA1,NDUFB3,NDUFA2,Atp5e,NDUFB9,PARK7,NDUFS6,NDUFS2,NDUFB6,ATP5MC3,COX4I1,ATP5PF,COX6B1,ATP5F1A,ATP5PO,UQCR11,NDUFA13,NDUFB11,NDUFS8,NDUFA11,UQCR10,NDUFB7,COX7A2,COX5A,NDUFA12
3	Sirtuin Signaling Pathway	16.9	0.12	NDUFA4,TIMM13,ATP5MC1,NDUFA7,TIMM10,ATP5F1D,NDUFB5,NDUFB8,HIF1A,NDUFA1,NDUFB3,NDUFA2,NDUFB9,TOMM7,SP1,ACSS2,NDUFS6,NDUFS2,NDUFB6,GSK3B,TP53,POLR1D,ATP5PF,ATP5F1A,TIMM8B,NDUFA13,Hist1h1e,NDUFB11,NDUFS8,NDUFA11,Tomm5,NDUFB7,TSPO,NDUFA12,H1F0
4	EIF2 Signaling	8.49	0.095	RPS19,ACTB,RPS18,EIF4A2,RPS15,WARS,RPL35,RPS16,RPL18A,RPS20,RPS26,PAIP1,RPS27L,SOS1,EIF3A,RPS25,GSK3B,RPS17,RPL18,RPL38,RPS14
5	Regulation of eIF4 and p70S6K Signaling	5.53	0.0892	RPS19,RPS18,EIF4A2,RPS15,RPS16,RPS20,RPS26,RPS27L,SOS1,PAIP1,EIF3A,RPS25,RPS17,RPS14
6	mTOR Signaling	4.31	0.0697	RPS19,RPS18,EIF4A2,HIF1A,RICTOR,RPS15,RPS16,RPS20,RPS26,RPS27L,EIF3A,RPS25,RPS17,RPS14
7	Aryl Hydrocarbon Receptor Signaling	3.3	0.0709	TGM2,TP53,RB1,TRIP11,GSTT2/GSTT2B,SP1,CCND3,MED1,NEDD8,GSTP1

Supplementary Table 5. Human antibodies used for mass cytometry analysis listing metal conjugate, antibody clone, supplier of each marker.

Isotope Tag	Antibody	Clone	Company	Catalogue nb
Y-89	CD45	HI30	Fluidigm	3089003B
Qdot 800 (Cd112/114)	CD14-Qdot	TüK4	Invitrogen	Q10064
Pd-102	Barcode (1:4000)			
Rh-103	Barcode (1:300)			
Pd-104	Barcode (1:2000)			
Pd-105	Barcode (1:100)			
Pd-106	Barcode (1:1000)			
Pd-108	Barcode (1:1000)			
Pd-110	Barcode (1:250)			
Pd-102	Barcode (1:4000)			
In-115	CD57	HCD57	Biolegend	322302
La-139	TCRγδ PE (Primary)	5A6.E9	Invitrogen	MHGD04
	Anti-PE (secondary)	PE001	Biolegend	408102
Ce-140	CD3	UCHT1	Biolegend	300402
Pr-141	HLA-DR	L243	Biolegend	307602
Nd-142	CLA	HECA-452	Biolegend	321302
Nd-143	Vα7.2	3C10	Biolegend	351702
Nd-144	Granzyme B	CLB-GB11	Abcam	ab103159
Nd-145	CD45RA	HI100	Biolegend	304102
Nd-146	CD8α	SK1	Biolegend	344702
Sm-147	CD45RO	UCHL1	Biolegend	304202
Nd-148	Vδ2	B6	Biolegend	331402
Sm-149	CD4	SK3	Biolegend	344602
Nd-150	CD103	B-Ly7	Thermo Fisher	14-1038-82
Eu-151	CCR4	205410	R&D Systems	MAB1567
Sm-152	Ki-67	B56	BD Biosciences	556003
Eu-153	CD49a	TS2/7	Biolegend	328302

Sm-154	CCR10	314305	R&D Systems	MAB3478
Gd-155	Foxp3 Biotin	PCH101	Thermo Fisher	13-4776-82
Gd-156	CCR7	150503	R&D Systems	MAB197
Gd-157	CD27	LG.7F9	Thermo Fisher	14-0271-82
Gd-158	CD56	NCAM16.2	BD Biosciences	559043
Tb-159	ICOS	C398.4A	Biolegend	313502
Gd-160	PD-1	eBioJ105	Thermo Fisher	14-2799-80
Dy-161	Vδ1 FITC (Primary)	REA173	Miltenyi Biotec	130-122-285
	anti-FITC (Secondary)	FIT-22	Biolegend	408305
Dy-162	CD161	HP-3G10	Biolegend	339902
Dy-163	CXCR3	49801	R&D Systems	MAB160
Dy-164	CCR9	L053E8	Biolegend	358902
Ho-165	CD38	HIT2	Biolegend	303502
Er-166	CXCR5	RF8B2	BD Biosciences	552032
Er-167	CD49d	9F10	Biolegend	304302
Er-168	CCR2	K036C2	Biolegend	357202
Tm-169	CD25	M-A251	Biolegend	356102
Er-170	CCR6	G034E3	Biolegend	353401
Yb-171	Integrin β7	FIB504	Biolegend	321202
Yb-172	BCL-2	100	Biolegend	658701
Yb-173	CD19	HIB19	Biolegend	302202
Yb-174	CX3CR1	K0124E1	Biolegend	355702
Lu-175	CCR5	HEK/1/85a	BIO-RAD	MCA2175
Yb-176	CD127	A019D5	Biolegend	351302
Ir-191/193	DNA		Fluidigm	201192A
Pt-195	Cisplatin		Sigma-Aldrich	201064
Bi-209	CD16	3G8	Fluidigm	3209002B

Supplementary Table 6. Socio demographic information of DM cohort and respective treatments.

Characteristic	All Other Monotherapy (n=18)	Metformin (n=42)	P-Value
Age x̅ (SD)	70.8 (7.8)	68 (8)	0.2045
Duration of DM x̅ (SD)	14 (11)	8.5 (8.3)	0.06375
Fasting glucose level x̅ (SD)	8.2 (2.76)	7 (1.8)	0.09648
Ethnic group			
Chinese/Malay/Indian n (%)	16 (88.9)/1 (5.6)/1 (5.6)	28 (66.7)/6 (14.3)/8 (19)	0.2018
Sex			
Female/Male n (%)	10 (55.6)/8 (44.4)	29 (69)/13 (31)	0.3812
Smoking			
Ex/Non/Smoker n (%)	3 (16.7)/12 (66.7)/3 (16.7)	5 (11.9)/33 (78.6)/4 (9.5)	0.6055

* All monotherapy : Acarbose (n=1 (5.5%)), Glibenclamide (n=3 (16.6%)), Glipizide (n=10 (55.5%)), Insulin (n=3 (16.6%)), and Tolbutamide (n=1 (5.5%)).

** Age and duration of DM in years. Fasting glucose level in mmol/L.

[#] Mann-Whitney *U* test

[ψ] Chi square test

Supplementary Table 7. Reagents used in this study.

Reagent	Company	Catalog number
Dynabeads untouched CD4 mouse cells	Invitrogen	11415D
Dynabeads untouched CD8 mouse cells	eBioscience	11417D
TB10.4(4-11)	New England Peptide	N/A
ESAT-6(3-16)	New England Peptide	N/A
Brefeldin A	Biolegend	420601
BD Cytofix/Cytoperm	BD Biosciencies	554714
BD Perm/Wash	BD Biosciencies	554723
ACK lysing buffer	Lonza/Gibco	10-548E/A1049201
Monensin	Biolegend	420701
BD Cytofix/Cytoperm Plus with BD GolgiPlug	BD Bioscience	555028
PMA	SIGMA	P1585-1mg
Ionomycin	SIGMA	10634-1 mg
anti-mouse CD3	BD Bioscience	553057
anti-mouse CD28	BD Bioscience	553294
PPD	Statens Serum Institut	2390
BD Horizon Brilliant Stain Buffer	BD Bioscience	566349
purified anti-mouse CD16/32	Biolegend	101302
Paraformaldehyde 16% solution	Electron Microscopy Sciences	15710
Human TruStain FcX	Biolegend	422302
mouse $CD8^{+}$ T cell isolation Kit	Miltenyi Biotec	130-104-075
FAM-FLICA Caspase-1 Assay Kit #98	ImmunoChemistry Technologies	98
D-(+)-Glucose solution	SIGMA	G8769-100ML
Poly-L-lysine solution	SIGMA	P4707-50ml
Seahorse XF Cell Mito Stress Test Kit	Agilent	103015-100

Seahorse XF Palmitate-BSA FAO Substrate	Agilent	102720-100
(+)-Etomoxir sodium salt hydrate	SIGMA	E1905-5MG
TRIzol Reagent	Life technologies	15596018
RNeasy Mini Kit	Qiagen	74106
iScript Advanced cDNA Kit for RT-qPCR	Bio-Rad	172-5038
iQ SYBR Green Supermix	Bio-Rad	170-8882AP
Arcturus PicoPure RNA Isolation kit	Thermofisher	KIT0204
Ovalbumin EndoFit	Invivogen	vac-pova
hIL-2 IS premium grade	Miltenyi Biotec	130-097-744
Recombinant hIL-15	R&D	247-ILB-005/CF
1,1-Dimethylbiguanide hydrochloride (Metformin)	Sigma Aldrich	

Supplementary Table 8. Mouse antibodies and dyes used for flow cytometry listing conjugate, antibody clone, supplier and catalog number of each marker.

Antibody/ dye	Fluorochrome	Clone	Company	Catalog nb
CD8a	FITC	Ly-2	Biolegend	108706
Perforin	PE	eBioOMAK-D	eBioscience	12-9392-80
CD3	PerCP-Cy5.5	17A2	Biolegend	100218
CD62L	PE-Cy7	MEL-14	eBiosciences	25-0621-81
CD4	Alexa Fluor 700	GK1.5	Biolegend	100429
CD44	PE-Cy5	IM7	eBioscience	15-0441-02
TNF alpha	APC	MP6-XT22	Biolegend	506308
IFN-gamma	Brilliant Violet 421	XMG1.2	Biolegend	505830
Zombie Aqua Fixable Viability Kit	Live/Dead Fix Aqua	N/A	Biolegend	423102
LIVE/DEAD™ Fixable Aqua Dead Cell Stain Kit	Pacific orange	N/A	Life technologies	L34957
CD3	FITC	145-2C11	Biolegend	100306
CD4	Pacific blue	GK1.5	Biolegend	100428
CD8	APC-Cy7	53-6.7	Biolegend	100714
CD44	BV650	IM7	Biolegend	103049
CD62L	PE-Cy7	MEL-14	Biolegend	104418
CD45	Pacific Blue	30-F11	Biolegend	103126
CD4	PerCP-Cy5.5	GK1.5	Biolegend	100434
CD62L	PE-CF594	MEL-14	BD	562404
CXCR3	BV605	CXCR3-173	Biolegend	126523
CD45	BV605	30-F11	Biolegend	103140
CD3	Pacific blue	145-2C11	Biolegend	100214
CD45	FITC	30-F11	Biolegend	103107

CD45.1	BV711	A20	Biolegend	110739
CD4	PE	GK1.5	Biolegend	100408
CD3	BUV395	17A2	BD	740268
CD45.2	APC	104	eBioscience	17-0454-82
CD62L	AlexaFluor700	MEL-14	Biolegend	104426
CD44	PE-Cy7	IM7	Biolegend	103029
IFNg	APC	XMG1.2	Biolegend	505810
Isotyp Rat IgG1	APC	N/A	Biolegend	400412
Mitotracker Green	FITC	N/A	Invitrogen	M7514
TMRM	PE	N/A	Thermo Fisher	T668
2-NBDG	FITC	N/A	Life technologies	N13195

Supplementary Table 9. Number of transferred cells into the recipient mice in adoptive transfer experiments.

Experiment Related Figure	Transferred cells	Number of transferred cells
Figure 1B	$CD4^+$ T	1×10^6
Figure 1B	$CD8^+$ T	1×10^6
Figure 1C	$CD4^+$ T	7×10^5
Figure 1C	$CD8^+$ T	7×10^5
Figure 3E	$CD8^+$ T	1×10^6
Figure 4F,G	$CD8^+$ T_M	3×10^4
Figure 4H	$CD8^+$ T_M	1×10^5
Figure 4H	$CD8^+$ T_E	5×10^4
Figure 4I	$CD8^+$ T_M	1×10^5

Supplementary Table 10. Human antibodies used for flow cytometry listing conjugate, antibody clone, supplier and catalog number of each marker.

Antibody	Fluorochrome	Clone	Company	Catalog nb
CD34	PE	563	BD	550761
CD4	BV750	SK3	BD	566355
CD8	BV605	SK1	BD	564116
CD14	BV605	M5E2	BD	564054
TCR γ/δ	APC-R700	11F2	BD	657706
CD27	BV510	L128	BD	563092
CD45	BUV805	HI30	BD	564914
CxCR3	BV650	G025H7	Biolegend	353730
TCR Vδ2	BV711	B6	Biolegend	331412
CD3	BV570	UCHT1	Biolegend	300436
TCR Vα7.2	APC-Cy7	3C10	Biolegend	351714
CD45RO	PerCP/Cy5.5	UCHL1	Biolegend	304222
CD161	BV785	HP-3G10	Biolegend	339930
Live/Dead Fixable Blue Dead Cell Stain Kit	BUV496	N/A	Invitrogen	L34962

Supplementary Data files

Supplementary Data 1. 202 Differentially expressed genes between splenic $CD8^+$ T cells from *Cxcr3*$^{-/-}$ vs WT untreated mice. FOXO1 targets are depicted.

Supplementary Data 2. 267 Differentially expressed genes between splenic memory-like $CD8^+$ T cells from metformin-treated vs untreated mice.

Supplementary Data 3. 607 Differentially expressed genes between splenic memory-like $CD8^+$ T cells from metformin-treated BCG-vaccinated vs untreated BCG-vaccinated mice.
