# Supplementary material for: Metformin enhances anti-mycobacterial responses by educating CD8+ T-cell immunometabolic circuits
Source: Nat Commun. 2020 Oct 16;11:5225. doi: 10.1038/s41467-020-19095-z (PMC7567856; doi:10.1038/s41467-020-19095-z)
Supplement: Supplementary file 2 — Description of Additional Supplementary Files [file 41467_2020_19095_MOESM2_ESM.pdf]

Description of Additional Supplementary Files

File Name: Supplementary Data 1
Description: 202 Differentially expressed genes between splenic CD8+ T cells from Cxcr3-/- vs WT untreated mice. FOXO1 targets are depicted.

File Name: Supplementary Data 2
Description: 267 Differentially expressed genes between splenic memory-like CD8+ T cells from metformin-treated vs untreated mice.

File Name: Supplementary Data 3
Description: 607 Differentially expressed genes between splenic memory-like CD8+ T cells from metformin-treated BCG-vaccinated vs untreated BCG-vaccinated mice.
