# Supplementary material for: Metformin enhances anti-mycobacterial responses by educating CD8+ T-cell immunometabolic circuits
Source: Nat Commun. 2020 Oct 16;11:5225. doi: 10.1038/s41467-020-19095-z (PMC7567856; doi:10.1038/s41467-020-19095-z)
Supplement: Supplementary file 6 — Reporting Summary [file 41467_2020_19095_MOESM6_ESM.pdf]

nature research

Corresponding author(s): Amit Singhal

Last updated by author(s): Aug 30, 2020

Reporting Summary

Nature Research wishes to improve the reproducibility of the work that we publish. This form provides structure for consistency and transparency in reporting. For further information on Nature Research policies, see our Editorial Policies and the Editorial Policy Checklist.

Statistics

For all statistical analyses, confirm that the following items are present in the figure legend, table legend, main text, or Methods section.

n/a	Confirmed	
☐	☒	The exact sample size (n) for each experimental group/condition, given as a discrete number and unit of measurement
☒	☐	A statement on whether measurements were taken from distinct samples or whether the same sample was measured repeatedly
☐	☒	The statistical test(s) used AND whether they are one- or two-sided<br>*Only common tests should be described solely by name; describe more complex techniques in the Methods section.*
☐	☒	A description of all covariates tested
☒	☐	A description of any assumptions or corrections, such as tests of normality and adjustment for multiple comparisons
☐	☒	A full description of the statistical parameters including central tendency (e.g. means) or other basic estimates (e.g. regression coefficient) AND variation (e.g. standard deviation) or associated estimates of uncertainty (e.g. confidence intervals)
☐	☒	For null hypothesis testing, the test statistic (e.g. F, t, r) with confidence intervals, effect sizes, degrees of freedom and P value noted<br>*Give P values as exact values whenever suitable.*
☒	☐	For Bayesian analysis, information on the choice of priors and Markov chain Monte Carlo settings
☒	☐	For hierarchical and complex designs, identification of the appropriate level for tests and full reporting of outcomes
☒	☐	Estimates of effect sizes (e.g. Cohen's d, Pearson's r), indicating how they were calculated

Our web collection on statistics for biologists contains articles on many of the points above.

Software and code

Policy information about availability of computer code

Data collection	Flow cytometry data was collected using FACSDiva software version 8.0.1 (BD Biosciences). Mass Cytometry data was collected using CyTOF softare version 7.0 (Fluidigm).
Data analysis	Data analysis was performed with GraphPad Prism Software (GraphPad Software Inc., version 7.01). Flow cytometry data was analysed with FlowJo software (Tree Star, Inc., USA, version 10.3). From RNA sequencing data differentially expressed genes (DEGs) were detected using DESeq2 method (http://www.bioconductor.org/packages/release/bioc/html/DESeq2.html) running in R package (.version 3.3.3)

For manuscripts utilizing custom algorithms or software that are central to the research but not yet described in published literature, software must be made available to editors and reviewers. We strongly encourage code deposition in a community repository (e.g. GitHub). See the Nature Research guidelines for submitting code & software for further information.

Data

Policy information about availability of data

All manuscripts must include a data availability statement. This statement should provide the following information, where applicable:

- Accession codes, unique identifiers, or web links for publicly available datasets
- A list of figures that have associated raw data
- A description of any restrictions on data availability

RNA sequencing data is available at NCBI's Gene Expression Omnibus through GEO Series accession number GSE139948. Primary raw data related to flow cytometry (Figure 1, 3, 4 and 5) and mass cytometry (Figure 2 and 6) analysis are available on request.

April 2020

Field-specific reporting

Please select the one below that is the best fit for your research. If you are not sure, read the appropriate sections before making your selection.

☒ Life sciences ☐ Behavioural & social sciences ☐ Ecological, evolutionary & environmental sciences

For a reference copy of the document with all sections, see nature.com/documents/nr-reporting-summary-flat.pdf

Life sciences study design

All studies must disclose on these points even when the disclosure is negative.

Sample size	No statistical method was used to predetermine sample size.<br><br>Mouse sample size per group varied from n=4-8 in different experiments. For Guinea pigs experiment sample size was 6-8 / group. These sample sizes / group in TB studies have earlier shown by us and others to provide valuable information, which is statistically significant.<br><br>Human study presented in Figure 6a-f, was from cohort of healthy subjects who received metformin (Approval #NL47793.091.14). In this cohort total 10 subjects were enrolled, however samples (pre- and post-metformin) were available from only eight subjects. We have earlier used samples from these subjects to get a clinically relevant information i.e. effect of metformin on monocytes (ref. 46, Lachmandas et al., 2019).<br><br>Data on diabetic subjects (Figure 6g) was mined from an observational study (NUS-IRB#04-140). Data of al subjects who were either on metformin mono-therapy (N=42) or other mono-therapies (N=18)were included in the present work.
Data exclusions	All data points have been included
Replication	Mouse experiments (8-28 mice/experimment), except for CyTOF analysis, were repeated at least two times. All replication were successful.<br>Guniea pig experiment (N=27 animals) was conducted once.<br>Ex vivo OT-1 experiment (Supplementary Fig 3) was conducted two times and data was reproducible.
Randomization	For each animal experiment, animals were randomly distributed into different groups at the beginning of the experiment. For Mtb infection studies, animals were randomly distributed into different groups post infection.<br>For healthy human sy=tudy (Figure 6a-f), subjects were randomized into two groups before study.<br>For diabetic cohort (Figure 6g), randomization was not relevant.
Blinding	Investigators were not blinded to allocation of different groups of animals. The clinical studies were not set up as blinded study.

Reporting for specific materials, systems and methods

We require information from authors about some types of materials, experimental systems and methods used in many studies. Here, indicate whether each material, system or method listed is relevant to your study. If you are not sure if a list item applies to your research, read the appropriate section before selecting a response.

Materials & experimental systems

n/a	Involved in the study	
☐	☒	Antibodies
☒	☐	Eukaryotic cell lines
☒	☐	Palaeontology and archaeology
☐	☒	Animals and other organisms
☐	☒	Human research participants
☒	☐	Clinical data
☒	☐	Dual use research of concern

Methods

n/a	Involved in the study	
☒	☐	ChIP-seq
☐	☒	Flow cytometry
☒	☐	MRI-based neuroimaging

Antibodies

Antibodies used	Antibodies for Mass Cytometry - Please refer to Supplementary Table 1 (human antibodies)and Supplementary Table 8 (for mouse antibodies).<br>Antibodies for Flow Cytometry - Please refer to Supplementary Table 13 (human antibodies) and Supplementary Table 11 (for mouse antibodies).
Validation	Each antibody was validated for flow Cytometry staining in previous publications and as well in the vendors website. Fluorescence-minus-one (FMO) and isotype controls were used during validation. Mass Cytometry antibodies have been validated and standardized by SIgN's CyTOF platform (Co author Evan Newell), and have been used in many publications before (One can refer to

Ref no. 24 and 25 for example).

Animals and other organisms

Policy information about studies involving animals; ARRIVE guidelines recommended for reporting animal research

Laboratory animals	8-12 weeks old SPF Female C57BL/6J (CD45.2), CD45.1, Cxcr3-/-, TCRbd-/- and OT-I mice were used. Hartley strain female guinea pigs aged between 7-8 weeks (350 – 400 g) were used. Animals were housed in the animal facilities on a 12/12h light/dark cycle, with food and water ad libitum.
Wild animals	No wild animals were used in the study.
Field-collected samples	No field collected samples were used in the study.
Ethics oversight	The study was approved by the Institutional Biosafety Committee (IBC) and Institutional Animal Care and Use Committee (IACUC) of the (i) Biological Resource Council (BRC), A*STAR, Singapore; (ii) Defence Science Organization (DSO) national laboratories, Singapore, (iii) Colorado State University, USA, and (iv) University of Massachusetts Medical School (UMMS), USA.

Note that full information on the approval of the study protocol must also be provided in the manuscript.

Human research participants

Policy information about studies involving human research participants

Population characteristics	In diabetic cohort the median age of subjects in two groups (See Supplementary Table 9) were 60-70 years old and involved 31-44% of males. The subjects in healthy participant group were Dutch adults (mean age 21 years) and included both males and females equally.
Recruitment	Healthy Dutch adults were enrolled in the study based on no self reporting underlying clinical conditions. They were given metformin in increasing doses ending with a commonly used dose of 1000 mg twice a day (see Reference 46, Lachmandas et al., 2019).<br><br>T2D patients included in the study are part of the Singapore Longitudinal Aging study 2 (SLAS-2), which is a population-based cohort intended to study the biology of aging among Singaporean elderly individuals above the age of 55 years old. They were enrolled upon their visit to endocrinology clinic at NUH.
Ethics oversight	The study in human healthy volunteers and diabetic patients was approved by the Arnhem-Nijmegen Ethical Committee (NL47793.091.14) and National University of Singapore Institutional Review Board (NUS-IRB #04-140), respectively.

Note that full information on the approval of the study protocol must also be provided in the manuscript.

Flow Cytometry

Plots

Confirm that:

- [x] The axis labels state the marker and fluorochrome used (e.g. CD4-FITC).
- [x] The axis scales are clearly visible. Include numbers along axes only for bottom left plot of group (a 'group' is an analysis of identical markers).
- [x] All plots are contour plots with outliers or pseudocolor plots.
- [x] A numerical value for number of cells or percentage (with statistics) is provided.

Methodology

Sample preparation	Single cell suspensions of spleens, lungs and lymph nodes (from mice) were prepared at the indicated time-points. Live dead staining was performed for 15 minutes at room temperature or for 20 minutes at 37°C. Non-specific antibody binding was blocked by adding anti-CD16/32 (Fc-block) (BD Biosciences). Following this surface markers were stained for 20-25 minutes at 4°C with the anti-mouse antibodies. In some experiments, mitochondrial staining was performed with 200 nM Mitotracker green and/or 20 µM Tetramethylrhodamine, methyl ester (TMRM) in RPMI media without FCS for 30 minutes at 37°C prior to staining of surface markers. Upon this cells were fixed in 2 % paraformaldehyde before acquisition.<br><br>For intracellular cytokine staining, spleen cells were ex vivo stimulated for 20 h with 40 µg/ml PPD (purified protein derivative, Statens Serum Institute, Denmark) in the presence of Golgi StopTM (BD Biosciences) and Monensin (1000x, BioLegend) for the last 4 hours, at 37°C and 5 % CO2. In some experiment's spleen cells were ex vivo stimulated with IL-2 and TB10.4 peptides for 5 hours at 37°C. Brefeldin A was added to the stimulation for the last 4 hours. Live dead staining and blocking was performed. Cells were then stained with respective surface antibodies. After staining, cells were washed, fixed and permeabilized for 20 minutes with Cytofix/ CytopermTM (BD Biosciences). Permeabilized cells were washed with Perm/ Wash bufferTM (BD Biosciences) and stained with antibodies against intracellular cytokines for 25 minutes at 4°C. Subsequently, cells were fixed in 2 % paraformaldehyde before acquisition.
Instrument	LSRII cytometer with 5 lasers (BD, San Jose, CA, USA) or Fortessa cytometer with 4 lasers (Becton Dickinson, San Jose, CA,

	USA) or Symphony cytometer (BD Biosciences, California, USA).
Software	FlowJo software, version 10.3 (Tree Star, Inc., USA)
Cell population abundance	MACS sorted CD4+ and CD8+ T cells showed purity >90%.
Gating strategy	CD8+ T cells: FSC/SSC, live/Dead, CD3+, CD8+<br>Memory CD8+ T cells: SC/SSC, live/Dead, CD3+, CD8+, CD62L+CD44+<br>For detailed gating strategies see Supplementary Fig 1a, c; Supplementary Fig 2a; Supplementary Fig 4a; Supplementary Fig 5a; and Supplementary Fig 7.

☒ Tick this box to confirm that a figure exemplifying the gating strategy is provided in the Supplementary Information.
